# Applications and development of in situ nucleic acid visualization techniques

**DOI:** 10.3389/fbioe.2026.1858210

**Published:** 2026-08-25

**Authors:** Sishuo Chen, Dahao Wei

**Affiliations:** 1 School of Nursing and Rehabilitation, Guangdong Chaozhou Health Vocational College, Chaozhou, Guangdong, China; 2 Key Laboratory of Systems Health Science of Zhejiang Province, School of Life Science, Hangzhou for Advanced Study, University of Chinese Academy of Sciences, Hangzhou, China

**Keywords:** FISH, HCR, in situ nucleic acid visualization, in situ sequencing, RCA

## Abstract

*In situ* nucleic acid visualization techniques are important tools and methods for studying the functions of nucleic acid molecules (DNA and RNA) and are widely used in various fields, including the spatial localization of gene expression, the influence of nucleic acid molecules on biological processes, and pathological diagnosis. On the basis of their mechanisms, these techniques can be categorized into direct detection methods, such as fluorescence *in situ* hybridization signal amplification-based techniques, such as Hybridization Chain Reaction (HCR), rolling circle amplification (RCA) and RNAscope; and combined coding and high-throughput imaging approaches, such as sequential Fluorescence *In Situ* Hybridization (seqFISH). In this review, we have established a comprehensive classification framework based on the fundamental principles of probe design and the mechanisms of signal amplification. We conducted an in-depth comparison of the strengths and limitations of *in situ* visualization methodologies in terms of spatial resolution, target throughput, and clinical applications. Furthermore, we explored potential major challenges in the development of *in situ* nucleic acid visualization technologies and proposed potential optimization strategies to address them. Finally, we looked ahead to the future directions for the expansion and development of *in situ* nucleic acid visualization technologies and analyzed their unique value in clinical translation.

## 1 Introduction

Cellular heterogeneity and the spatial distribution of cells within tissues determine the diverse biological functions they perform. Although traditional transcriptomic sequencing and single-cell transcriptomics technologies can effectively elucidate cellular composition and corresponding molecular mechanisms, these methods cannot capture spatial information, such as the tumor immune microenvironment, neural synaptic connections, and key spatial signals in embryonic polarity development (Gulati et al., 2025; Author anonymous, 2023; Sorin et al., 2023). Consequently, the development of *in situ* nucleic acid visualization techniques capable of providing both molecular quantification and *in situ* information has become central to current spatial omics research.

The exploration of *in situ* nucleic acid visualization began with the development of FISH technology. Early FISH primarily addressed the basic needs for the identification and localization of target genes within the broader genomic context’, since early FISH probes were relatively large and therefore commonly targeted genes together with adjacent genomic regions rather than single genes alone (Swiger and Tucker, 1996; Huber et al., 2018). Understanding the three-dimensional interactions of functional elements on the genome is of great significance for elucidating gene regulation. Traditional proximity-linking techniques, such as Chromosome Conformation Capture (3C), Circular Chromosome Conformation Capture (4C), Chromosome Conformation Capture Carbon Copy (5C), and Chromatin Interaction Analysis by Paired-End Tag sequencing (ChIA-PET), primarily rely on the use of formaldehyde to cross-link and immobilize spatially adjacent DNA and protein complexes (Dekker et al., 2002; Zhao et al., 2006; Dostie et al., 2006; Fullwood et al., 2009). However, it is worth noting that these methods typically cannot provide unbiased genome-wide spatial maps. 3C is primarily designed for one-to-one analysis, 4C for one-to-many analysis, and 5C for many-to-many analysis, but all rely on pre-designed primers (Simonis et al., 2006). Building upon existing technologies, High-throughput Chromosome Conformation Capture (Hi-C) represents an upgrade to 3C technology and is currently the primary detection method in 3D genomics. However, it also suffers from high background noise, and Hi-C struggles to directly distinguish whether three or more fragments are part of the same complex or represent independent pairwise interactions occurring in different cells (Lieberman-Aiden et al., 2009; Rao et al., 2014). Split-Pool Recognition of Interactions by Tag Extension (SPRITE) technology represents a further advancement over Hi-C, it does not require DNA fragment ligation but instead utilizes DNA barcoding for labeling. Its primary advantages include the ability to simultaneously detect multiple (ranging from a few to thousands) DNA fragments within a single complex, while eliminating ligation-related false positives and significantly reducing background noise. However, it suffers from relatively poor resolution for short-range interactions and involves a relatively cumbersome experimental workflow (Quinodoz et al., 2018; Arrastia et al., 2022).

Nevertheless, although the aforementioned sequencing-based analyses of three-dimensional gene-space interactions offer the advantage of high throughput, they fundamentally rely on cell lysis products for analysis. They cannot preserve the original morphological context of cells, making it difficult to fully capture spatial heterogeneity at the single-cell level. To address this practical issue, *in situ* nucleic acid visualization techniques, represented by FISH, remain indispensable. However, when low-abundance transcripts such as certain long noncoding RNAs (lncRNAs) including XIST and MALAT1 are detected or signals are extracted from complex tissues, traditional FISH often faces issues of insufficient sensitivity and high background noise (Choudhury et al., 2024; Cabili et al., 2015). To address these challenges, strategies for *in situ* signal amplification have garnered significant attention. Among these, the hybridization chain reaction (HCR) achieves linear amplification of *in situ* signals through the self-assembly of DNA hairpin structures. The latest version (v3.0) demonstrated superior performance in terms of the signal-to-noise ratio and tissue penetration and have been widely applied in imaging vertebrate embryos and brain tissues (Schulte et al., 2024; Choi et al., 2018; Borum and Jokerst, 2021). Moreover, rolling circle amplification (RCA), leveraging the high persistence of the polymerase, generates extremely intense fluorescent signal spots. Additionally, owing to its locked-loop probe characteristics, it demonstrates excellent performance in the *in situ* detection of single nucleotide polymorphism (SNP) and low-abundance transcripts (Kalhor et al., 2024; Stower, 2013; Deng et al., 2017; Gu et al., 2018). In addition, *in situ* loop-mediated isothermal amplification (IS-LAMP) is another nucleic acid visualization technique worthy of attention. The core IS-LAMP technology relies on a DNA polymerase with strong strand-displacement activity and multiple target-specific primers to amplify DNA under isothermal conditions (Wang T. et al., 2025). These *in situ* amplification techniques not only standardize single-molecule detection but also provide core support for the development of subsequent high-throughput *in situ* sequencing and three-dimensional single-nucleotide imaging technologies.

**FIGURE 1:**
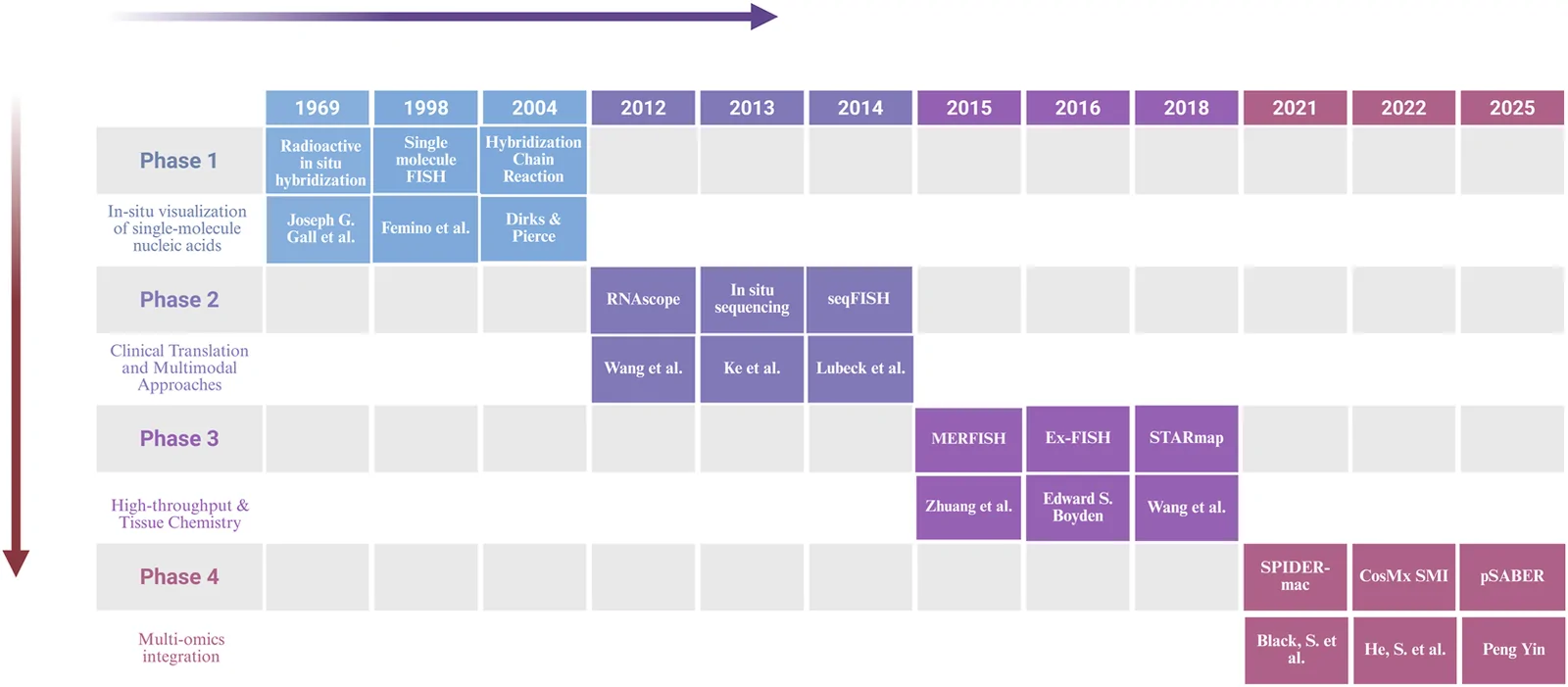
Development of *in situ* nucleic acid visualization technology. Created with Biorender.com.

As shown in Figure 1, in situ nucleic acid visualization has gone through several distinct stages of development. As exploration of critical life science questions continues, single-molecule *in situ* imaging technology is no longer sufficient to address complex scientific problems. For instance, regarding spatial heterogeneity in tumor clonal evolution, traditional transcriptomics and single-molecule imaging technologies cannot resolve differences in transcriptional lineages across distinct regions within a tumor lesion. Furthermore, they are unable to reveal issues such as physical selective pressures related to the degree of surrounding stromal fibrosis and gradients in vascular density (Arora et al., 2023; Venning et al., 2021; Mo et al., 2024; Williams et al., 2026). Consequently, building upon the aforementioned *in situ* hybridization and signal amplification technologies and driven by the rapid development and integration of bioinformatics and artificial intelligence, research on nucleic acid *in situ* visualization has shifted from single-molecule *in situ* analysis to whole-transcriptome *in situ* analysis. Hybrid coding imaging technologies, such as Multiplexed Error-Robust Fluorescence *In Situ* Hybridization (MERFISH) and seqFISH+, enable the simultaneous detection of the expression of thousands of genes at the single-cell level through multiround hybridization (Zou et al., 2025; Choi et al., 2023; Watson et al., 2025; Cilento et al., 2024). Concurrently, *in situ* sequencing technologies, such as Hybridization-based *In Situ* Sequencing (HybISS) and Spatially-resolved Transcript Amplicon Readout mapping (STARmap), have achieved a high degree of integration between molecular information and anatomical structure by directly reading sequence data *in situ* within tissues (Zeng et al., 2023a; Wu et al., 2024; Gyllborg et al., 2020; Wang X. et al., 2018). Currently, *in situ* nucleic acid visualization techniques are evolving toward three-dimensional single-molecule nucleic acid imaging. By combining histochemical clearing with expansion microscopy, researchers have achieved single-molecule localization in thick tissue sections and partially intact organs (such as the entire mouse brain) at nanoscale resolution (Gandin et al., 2025; Alon et al., 2021; Wang et al., 2021).

In clinical research, the combination of *in situ* nucleic acid visualization technology and deep learning is continuously expanding the horizons of spatial digital pathology. In particular, advances in in situ nucleic acid visualization technology have improved our understanding of the tumor immune microenvironment, enabling researchers to discover entirely new mechanisms of immune regulation, such as those involving tertiary lymphoid structures (Tang et al., 2025; Wang X. et al., 2024; Wang Y. et al., 2025; Sautès-Fridman et al., 2019; Li et al., 2025; Hoang et al., 2024; Fu et al., 2025; Roetzer-Pejrimovsky et al., 2024). Concurrently, these advancements have driven the evolution of companion diagnostics. Through in-depth analysis of the localization of various immune markers—such as CK, CD8, CD68, PD-1, and PD-L1—in patient tissue sections, it has become possible to generate immune scores for patients, thereby facilitating the selection of appropriate immunotherapies (Ghiringhelli et al., 2023; Aung et al., 2025; Prabhakaran et al., 2026; Wang et al., 2022).

This article provides a systematic review of the evolution of *in situ* nucleic acid visualization technologies, focusing on three key dimensions: detection sensitivity, multiplexing capabilities, and complex spatial data analysis. It delves into the necessity of technologies such as HCR, RCA, and *in situ* sequencing in addressing current biological challenges and outlines their application boundaries and future trends in clinical diagnostics.

## 2 Technical principles

### 2.1 *In situ* nucleic acid visualization based on direct detection methods

**FIGURE 2:**
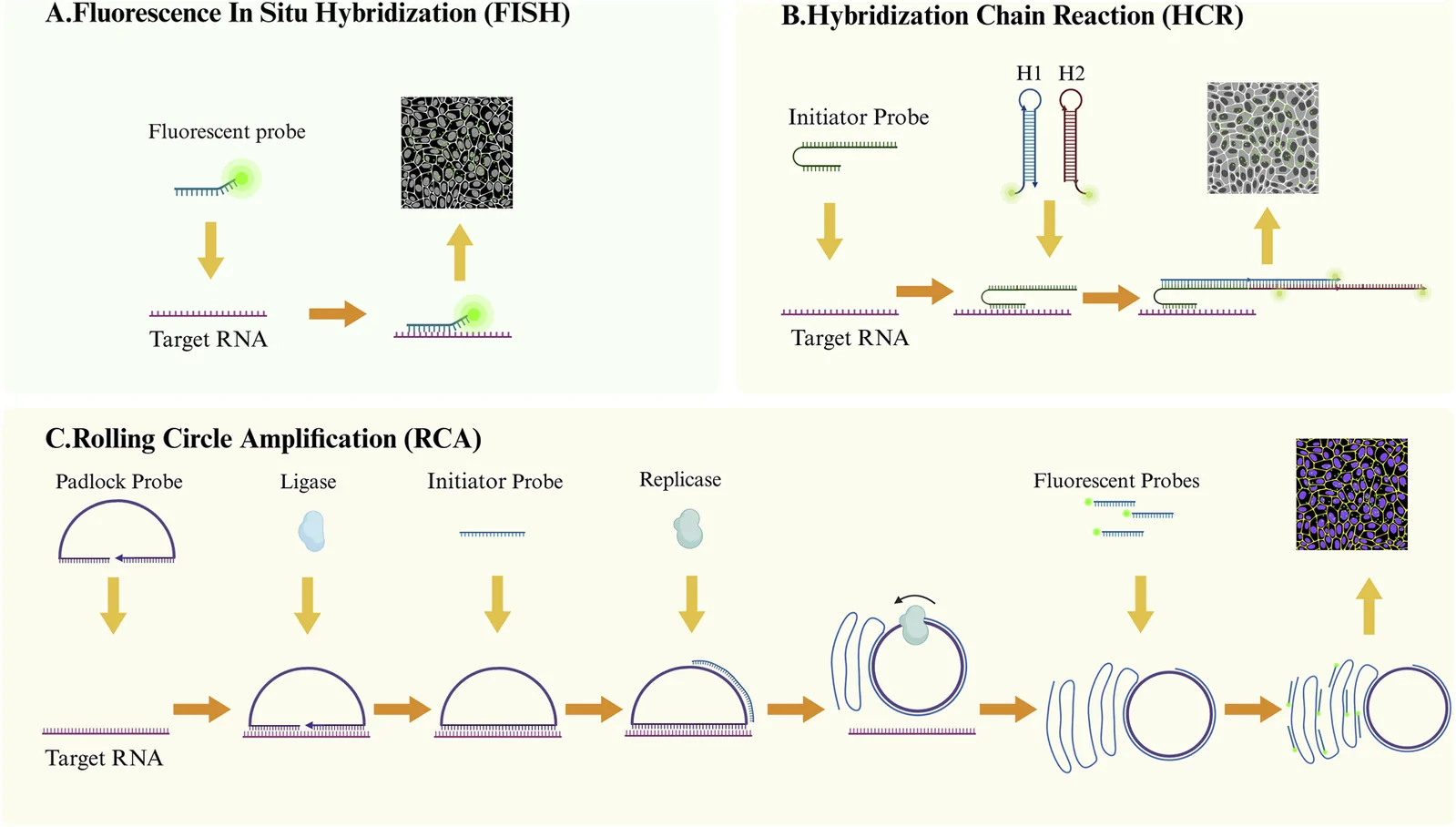
Principles of three nucleic acid *in situ* visualization techniques. **(A)** Principle of Fluorescence *In Situ* Hybridization. **(B)** Principle of Hybridization Chain Reaction. **(C)** Principle of Rolling Circle Amplification Reaction. Created with Biorender.com.

The concept of FISH was initially pioneered by Bauman et al., in 1980, who successfully localized specific DNA sequences using fluorochrome-labeled RNA probes. Subsequently, the technique evolved with the development of DNA-based probes, which have since become the standard for modern FISH applications (Bauman et al., 1980). This direct detection method involves designing oligonucleotide probes that can form base-pairing complementary bonds with the target nucleic acids, labeling them with a fluorophore, and finally achieving fluorescence enrichment through the hybridization and binding of the oligonucleotide probes to the target nucleic acids, thereby detecting these nucleic acids (Figure 2A). In response to the demand for higher precision in single-molecule imaging, Raj A. et al. developed single-molecule FISH (smFISH), a technique capable of *in situ* identification of individual mRNAs, revealing the phenomenon of localized translation of mRNAs in neuronal dendrites (Raj et al., 2008; Buxbaum et al., 2015). After decades of optimization, the experimental workflow of FISH has become highly standardized, enabling the identification and analysis of various tissue samples. It serves as a crucial tool for the clinical detection of chromosomal translocations (e.g., ALK) and gene amplification (e.g., HER) (Hendriks et al., 2023; Adesoye et al., 2026; Press et al., 2016). Notably, in the *in situ* detection of DNA on chromosomes, traditional probe preparation methods rely primarily on the BAC method (Zhang J. et al., 2023),however, Wu et al. pioneered the development of Oligopaint, marking the official transition of FISH technology from traditional large-fragment cloned probes (BAC probes) to the era of highly programmable oligonucleotide probes. The research team revolutionized FISH probes through two key aspects: specificity optimization and sensitivity enhancement. To address specificity issues, Wu et al. achieved single-copy accuracy and thermodynamic uniformity through *in silico* analysis. Using bioinformatics algorithms, the research team identified potential duplicate sequences and homologous pseudogene sequences at the computational level through rigorous k-mer filtering and BLAST alignment. Additionally, to improve hybridization efficiency, Oligopaint’s algorithm strictly limits the GC content and melting temperature (Tm) of all probes, ensuring that different probes targeting the same gene bind non-specifically to nucleic acids uniformly under the same conditions, thereby reducing the risk of mismatch-induced off-target binding (Beliveau et al., 2012). Although Classical FISH is generally simple and straightforward, certain limitations remain, such as insufficient detection sensitivity, particularly for low-abundance transcripts, including certain long noncoding RNA such as MALAT1 and XIST (Soares et al., 2018; Zhu et al., 2023). Additionally, when samples with strong autofluorescence (such as paraffin-embedded tissues) are analyzed, Classical FISH results are often compromised (Cui et al., 2018; Yang et al., 2024; Croce and Bottiroli, 2014; Lin et al., 2010; Dalal and Slinger, 1985). When probes for target nucleic acids are designed, the target nucleic acid must be of sufficient length (>1 kb) to accommodate approximately 30–48 oligonucleotide probe sites to form a visible spot; this makes localization analysis difficult for some short RNAs, such as miRNAs (22 nt) and piRNAs (26–31 nt) (Robles-Re et al., 2025; Lu and Tsourkas, 2009). To address this issue, Clemens Steinek et al. developed a technique using densely labeled oligonucleotides to detect small genomic elements, enabling *in situ* hybridization of non-repetitive sequences smaller than 1 kb, such as micro-enhancers. By utilizing screened DNA polymerases to efficiently incorporate fluorophore-labeled nucleotides into DNA strands, and by optimizing primer design and enzymatic reaction conditions, the researchers successfully synthesized oligonucleotide probes densely labeled with fluorescent molecules. The introduction of this method addresses the challenge of incorporating more fluorescent groups within the limited probe design space (Steinek et al., 2024).

It is worth noting that when performing FISH experiments on tissue sections or cell samples, standard FISH procedures often cause significant damage to cell morphology and chromatin structure. First, during the sample preparation stage, prior to formal *in situ* hybridization, tissue sections or cell samples must undergo decarboxylation and deproteinization to facilitate the entry of macromolecular probes and better expose the probe-binding sites on the target nucleic acid molecules. This step typically involves treatment with detergents such as Triton X-100, acid treatment (0.1 M HCl), and protease treatment (proteinase K or pepsin). While these steps significantly increase the success rate of probe binding, they also cause irreversible structural damage, such as loosening or displacement of chromatin structures. During *in situ* hybridization, thermal denaturation (70 °C–80 °C) and the addition of high-concentration denaturants (formamide) are often required to maintain the double-stranded structure of DNA and expose probe binding sites. This treatment with high temperatures and chemical reagents frequently causes deformation of the cell nucleus and disrupts the fine topological structure of chromatin at the nanoscale (e.g., TADs and chromatin loops). Additionally, during the final washing steps, a salt concentration gradient is often used to remove non-specifically bound probes; this change in osmotic pressure can also affect cellular structure (Solovei et al., 2002; Markaki et al., 2013).

To address the issue of how tissue clearing and deproteinization affect intracellular structures, Chen, F., et al. proposed a tissue clearing technique using hydrogels (Hydrogel Embedding/ExFISH). Prior to tissue clearing or deproteinization, the sample is treated with acrylamide monomers. These monomers chemically cross-link with intracellular nucleic acids, forming a three-dimensional hydrogel network within the cell. During subsequent processing, even as the supporting proteins are digested, this network maintains the three-dimensional spatial structure of the nucleic acids (Chen et al., 2016). Additionally, a strategy combining ultra-short probes with mild permeabilizing agents can reduce the need for sample permeabilization steps. For example, designing extremely short single-stranded DNA probes using the OligoPaints probe library, or substituting Triton X-100 or proteases with digitonin, can achieve the same effect (Beliveau et al., 2012). To address the issue of thermal denaturation during *in situ* hybridization, the RASER-FISH (single-strand excision nucleotidase-mediated FISH) technique proposed by Brown, J. M., et al. can be employed (BrdU/BrdC, thymidine analogs, are added during the cell culture stage to incorporate them into newly synthesized DNA). After cell fixation, irradiation with long-wavelength ultraviolet (UV) light creates a nick at the BrdU site. Upon addition of exonuclease III (Exo III), the enzyme degrades one strand of DNA along the nick, ultimately yielding a single strand) (Brown et al., 2022). To address the issue of cell structure disruption caused by washing in a salt concentration gradient, one can employ an *in situ* hybridization strategy based on molecular beacons or hairpin-like probes, utilizing the principle of proximity quenching between a fluorescent group and a quencher to generate a specific fluorescent signal (Tyagi and Kramer, 1996).

### 2.2 *In situ* nucleic acid visualization based on signal amplification strategies

*In situ* nucleic acid visualization technologies based on signal amplification strategies can be divided into two main categories: enzymatic amplification and enzymatic-free self-assembly. Rolling circle amplification (RCA) is a mainstream enzymatic signal amplification reaction. Upon binding to lock-on probes, it enables the localization and analysis of nucleic acid molecules (Figure 2C). The core of RCA lies in the design of the ligated probes. Only when the 5′ and 3′ sequences of the ligated probe strictly bind to the corresponding sites on the target nucleic acid can they be linked by a ligase for subsequent reactions. Due to this characteristic, RCA can be applied to the *in situ* detection of single nucleotide polymorphisms (SNPs) (Nilsson et al., 1997). The primary advantages of RCA-based *in situ* nucleic acid visualization technology lie in its extremely high sensitivity and signal amplification capabilities. Owing to the characteristics of the RCA reaction, it can generate hundreds to thousands of duplicate copies at a single binding site on the nucleic acid to enrich fluorescent molecules (Zhong et al., 2001; Ali et al., 2014). Unlike FISH, the reaction does not require target nucleic acid molecules larger than 1 kb, enabling *in situ* imaging analysis of shorter nucleic acid molecules. Additionally, the nanospheres generated by RCA can be fixed within the tissue; compared with the linear products produced by HCR, they excellently preserve the spatial positioning information of transcripts (Lee et al., 2014). However, it is worth noting that the RCA reaction steps are relatively cumbersome compared with those of FISH and HCR. The nanospheres produced by the reaction are too large, which can easily lead to molecular crowding when highly expressed transcripts are detected or when multiple transcripts are codetected, thereby limiting the RCA reaction (Park et al., 2025; Mi et al., 2025).

HCR is a classic self-assembly based signal amplification strategy proposed by Robert M. Dirks and Niles A. Pierce in 2004. It is a reaction system composed of a pair of metastable hairpin-structured nucleic acids and an initiator strand (Dirks and Pierce, 2004). The designers ingeniously applied the principle of complementary base pairing to design two single-stranded DNA molecules such that they form stable hairpin structures at appropriate temperatures and salt concentrations (Figure 2B). However, when the hairpin structures open, the two strands can hybridize, producing a cascade amplification effect. The basic components of the Hybridization Chain Reaction include two hairpin nucleic acids (H1 and H2) and an initiator strand (I-strand). Compared with *in situ* nucleic acid visualization techniques based on enzyme-mediated signal amplification, HCR offers a simpler experimental procedure because its amplification is driven by the hybridization kinetics of metastable hairpin structures. The hairpin-structured nucleic acid molecules used in HCR typically have a molecular weight of 30–50 nt. Compared with the 100–120 nt lock-on probes and polymerases used in RCA, these smaller molecules exhibit better tissue penetration. This enables more uniform reactions throughout both the interior and exterior of thick tissue sections and intact tissues during *in situ* nucleic acid analysis (Choi et al., 2018; Choi et al., 2014; May-Zhang et al., 2022; Choi et al., 2010). S Shigeaki Kanatani et al. reported that adjusting the HCR temperature to 4 °C enables consistent reaction levels both inside and outside intact tissue (Kanatani et al., 2024). Concurrently, with the advent of HCR v3.0, the use of cleavable probes to initiate the HCR has enabled HCR-based *in situ* nucleic acid visualization technology to achieve single-molecule quantitative analysis (Choi et al., 2018). However, because the nucleic acid molecules in the HCR products are noncovalently bound, care must be taken in terms of the washing conditions during sample processing; furthermore, the stability of its products is inferior to that of RCA.

In addition to HCR, a self-assembly based signal amplification system, the RNAscope technology developed by Advanced Cell Diagnostics (ACD) represents another mainstream self-assembly based *in situ* nucleic acid visualization system. On the basis of a unique double-Z-shaped probe design, fluorescence enrichment strategies, and standardized experimental protocols, it has become a mainstream method for the *in situ* detection of RNA and DNA (Wang et al., 2012; Atout et al., 2022; Mead and Apte, 2020; Deleage et al., 2018).

The core of the RNAscope technology lies in its dual Z-probe design. Only when two adjacent Z-probes bind simultaneously to the target RNA can subsequent probes bind and initiate the signal amplification reaction. If only one Z-probe binds, the amplification process cannot be initiated. This ingenious design effectively eliminates background noise and non-specific binding. Once the dual Z-probes bind, the Pre-amplifier, Amplifier, and Label probe are sequentially recruited, ultimately forming a signal-enriched molecule that achieves signal amplification of approximately 8,000-fold or more (Wang et al., 2012) (Table 1). In clinical practice, the rNAscope technology demonstrates significant advantages, including FFPE compatibility, standardized testing procedures, and standardized diagnostic results. In the testing of FFPE samples, issues such as RNA sequence fragmentation and nucleic acid cross-linking frequently arise (Masuda et al., 1999; von Ahlfen et al., 2007). Due to this fragmentation and cross-linking, *in situ* nucleic acid visualization based on RCA may encounter breaks in the binding targets, preventing subsequent RCA reactions. Additionally, nucleic acid cross-linking in FFPE samples may affect the hybridization of the lock-in probes and subsequent enzymatic reactions, thereby impacting *in situ* hybridization (Ke et al., 2013). At the same time, the rNAscope technique addresses the limitations of immunohistochemistry (IHC) assays. For example, for disease biomarkers such as TGF-β (secreted protein) and LGR5 (G protein-coupled receptor), IHC detection is often too limited. However, with the help of the rNAscope technique, analysis can be performed by detecting their mRNA. At the same time, rNAscope has also demonstrated good performance in precisely locating viral infections in the tumor microenvironment, such as HPV and EBV (Wang et al., 2012; Baker et al., 2014).

**TABLE 1:** Comparison of three nucleic acid In situ visualization techniques.

Parameter	Classical FISH	HCR	RCA	RNAscope
Spot Size(nm)	∼50 nm (Subcellular localization)	∼250 nm (Subcellular localization)	∼100–300 nm (Subcellular localization)	∼200–300 nm (Subcellular localization)
Signal Gain	None	∼200 × 1,000× (Choi et al., 2018)	∼1,000 × 5,000× (Lizardi et al., 1998)	∼8,000×
Tissue Permeability	Excellent	Excellent	Generally	Excellent
Amplification Mechanism	None	Enzyme-free	Enzymatic	bDNA tree assembly
Multiplexing Capacity	Low (3–5)	Medium/High	Extremely High	Low (2–4 plex natively)
FFPE Compatibility	Poor	Good	Poor/Moderate	Excellent

In addition to *in situ* detection technologies for RNA, DNAscope offers high spatial resolution and signal-to-noise ratio for detecting gene amplifications and deletions in tumors. Furthermore, to prevent interference from mRNA in the tissue, samples undergo RNase treatment prior to DNAscope analysis to eliminate the impact of mRNA on the results (Deleage et al., 2016). In the *in situ* detection of short target nucleic acids (<1 kb), there may be a shortage of available sites for probe design. To address this issue, the Basescope technology utilizes multi-level amplification molecules to enable *in situ* visualization of nucleic acids with the binding of just a single pair of Z-probes. Furthermore, Basescope can also be applied to the detection and analysis of point mutations and alternative splicing (Nielsen et al., 2020; Baker and Graham, 2020) (Table 2).

**TABLE 2:** Comparison of RNAscope, DNAscope and BaseScope.

Parameter	RNAscope	DNAscope	BaseScope
Target Type	mRNA/lncRNA	Genomic DNA	Short Sequences/Splice Variants
Required Length of Target Nucleic Acid	>300 bp	>20 kb	50–300 bp
Magnification	∼8,000×	∼1,000×	>100,00×
Spatial Resolution	Subcellular	Chromosome/Locus	Single Molecule
FFPE Compatibility	Highly Suitable	Suitable	Suitable
Clinical Applications	Tumor Microenvironment/Biomarkers	Gene Amplification/Deletion	Exon Deletion/Point Mutation Testing

### 2.3 Combinatorial coding and high-throughput imaging

**FIGURE 3:**
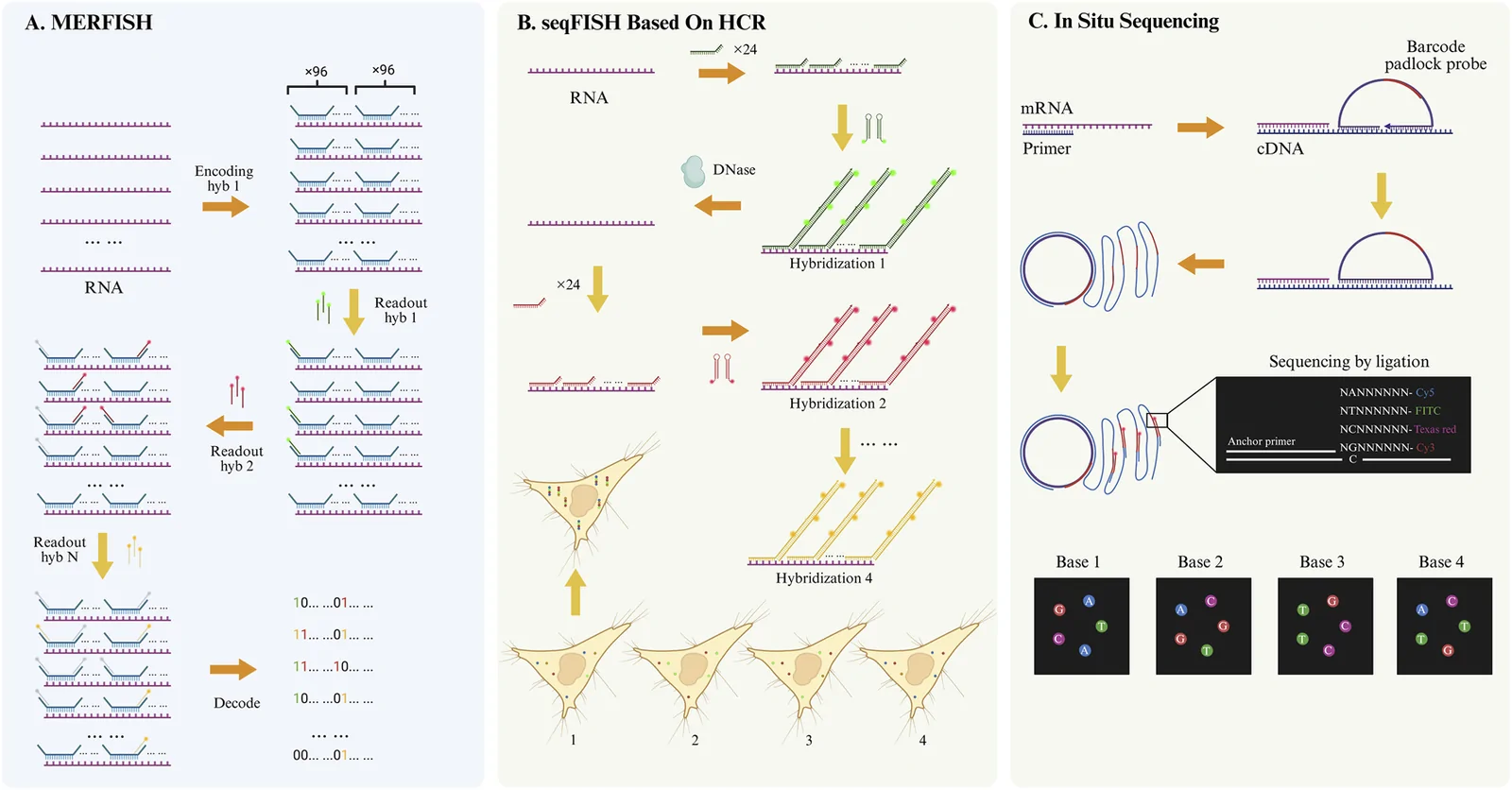
Principles of three classic *in situ* sequencing methods. **(A)** Principle of MERFISH *in situ* sequencing. **(B)** Principle of HCR-based *in situ* sequencing. **(C)** Principle of rolling circle amplification-based *in situ* sequencing. Created with Biorender.com.

Owing to spectral limitations, conventional *in situ* nucleic acid visualization techniques typically allow the use of only four fluorescent labels with significantly different wavelengths to identify target nucleic acids (Valm et al., 2016; Lubeck and Cai, 2012), which severely limits the ability to obtain more *in situ* information from the same tissue. Combination coding generates a corresponding barcode by analyzing the different fluorescent spots at the same location during each round of hybridization; by comparing these barcodes, the transcript information at that location can be analyzed (Chen et al., 2015; Eng et al., 2019; Rouhanifard et al., 2018; Dardani et al., 2022). Different FISH-based *in situ* sequencing methods employ distinct encoding and error-correction mechanisms. For example, seqFISH plus, developed by Long Cai et al., uses a palette encoding mechanism to assign 10,000 genes to different colors and hybridization rounds; however, it lacks an error-correction mechanism and relies solely on imaging at extremely high rounds to physically dilute the signal density in each round (Eng et al., 2019). In contrast, multiplexed error-robust FISH (MERFISH), developed by Zhuang Xiaowei et al., encodes RNA in a binary barcode format combined with Hamming distance coding. Even if false-positive or false-negative results occur in a given round of imaging, the algorithm can correct errors, thereby significantly improving the detection accuracy, with the detection efficiency reaching 80% (Figure 3A) (Chen et al., 2015; Moffitt et al., 2016). This technology has been commercialized and has become the core technology of Vizgen’s *in situ* sequencing. FISH-based *in situ* sequencing technologies often face challenges with respect to probe penetration efficiency when thick tissues are sequenced. Enhanced electric field FISH (EEL-FISH), developed by Linnarsson et al., incorporates the concept of electric field acceleration. The use of an electric field to drive negatively charged probes to rapidly penetrate thick tissue enables large-scale, high-throughput imaging at the centimeter level while significantly reducing sequencing time (Borm et al., 2023). Meanwhile, the Perturb-FISH technology developed by Loϊc Binan et al. enables large-scale spatial CRISPR screening based on *in situ* optical imaging. The main workflow includes Pooled CRISPR Perturbation, *In situ* Amplification, and Optical Readout *via* MERFISH. The core advantage of Perturb-FISH lies in its ability to preserve spatial information, unlike traditional screening techniques based on single-cell RNA sequencing. This makes Perturb-FISH extremely valuable for studies aimed at deciphering intercellular communication, analyzing native spatial morphology and tissue architecture, and revealing subcellular structure and microenvironment-dependent regulation (Binan et al., 2025).

Additionally, HCR-based *in situ* sequencing technology has demonstrated excellent performance in the *in situ* imaging analysis of thick tissue. Schwarzkopf, M., et al. developed DNA-barcoded HCR (dbHCR), which involves labeling each HCR amplification product with a unique DNA barcode and subsequently sequencing it to determine transcript information (Wang Y. et al., 2024). Currently, RCA-based *in situ* sequencing technology is widely used; for example, companies such as 10× Genomics and Cartana all employ this method (Gyllborg et al., 2020; Lee et al., 2014; Ke et al., 2013; Janesick et al., 2023). Unlike FISH-based *in situ* transcriptomic analysis, RCA-based *in situ* sequencing does not rely on fluorescence-coded readouts for interpretation; instead, it derives results through sequencing analysis of the base sequence barcodes in RCA products, offering the potential for higher throughput (Figure 3C) (Crosetto et al., 2015; Chen et al., 2018). Wang Xiao et al. developed STARmap technology, which enables large-scale three-dimensional *in situ* quantitative analysis of tissues. Furthermore, by bypassing reverse transcription and performing *in situ* analysis directly on mRNA, 3D transcriptomic analyses of neural circuits in brain tissue sections 100–500 μm thick can be performed (Wang X. et al., 2018). The team subsequently improved the design of RCA primer-triggered probes by adding ribosome-triggered probes, thereby achieving *in situ* sequencing analysis of the translome (Zeng et al., 2023b). For *in situ* sequencing, precision is as critical as throughput. A team led by Alon, S., et al. combined RCA with tissue physical expansion (ExM), expanding tissue 4–20-fold within a hydrogel prior to sequencing, thereby achieving molecular localization analysis with a resolution of 20 nm (Alon et al., 2021). In addition to focusing on various *in situ* sequencing technologies, auxiliary techniques such as micro-expansion microscopy are also being increasingly widely applied in in situ sequencing. The traditional ExM sample preparation process is time-consuming. The development of BOOST (microwave-accelerated rapid 10-fold expansion microscopy) technology has accelerated the processes of fixation, permeabilization, gel polymerization, and deproteinization. It enables single-step 10-fold expansion in a short time and can also be applied to FFPE sections and millimeter-scale tissue samples (Guo et al., 2025). To achieve higher magnification factors, the previous approach required Iterative ExM—that is, performing a second expansion after embedding following the first expansion—which was relatively cumbersome. To address this issue, Wang, S., et al. developed the Single-shot 20ExM technology. By optimizing the composition ratio of hydrogel monomers and crosslinkers, this technology achieves high physical magnification in a single-layer gel in a single step (Wang S. et al., 2024).

## 3 Potential challenges and optimization strategies of in situ nucleic acid visualization

### 3.1 Precision and optical crowding

In in situ nucleic acid visualization techniques, imaging precision and accuracy are central to achieving single-molecule resolution detection. However, as the detection throughput continues to increase, a severe optical crowding effect arises between the signal gain and the diffraction limit (Lubeck and Cai, 2012; Moffitt et al., 2016; Wang G. et al., 2018; Maniatis et al., 2021). Biologically, this phenomenon is particularly pronounced when high-abundance transcripts are detected. Dense probe hybridization causes signal points to merge in terms of their spatial distribution. From a technical perspective, signal amplification strategies further exacerbate this phenomenon. For example, in RCA-based *in situ* nucleic acid visualization imaging, the resulting DNA nanospheres typically have diameters of 200–400 nm; the high-density enrichment of fluorescent molecules creates a crowding effect that leads to signal overlap (Lee et al., 2014; Chang et al., 2023). Moreover, in HCR-based *in situ* nucleic acid visualization, the resulting linear double-stranded DNA products typically reach lengths of 500 nm–1 μm (Dirks and Pierce, 2004; Choi et al., 2010). This signal diffusion readily leads to cross-displacement artifacts across different mRNA molecules, severely compromising subcellular localization and quantitative analysis.

To address the trade-off between signal intensity and spatial resolution, researchers have developed multidimensional optimization strategies. To address signal drift caused by excessively long HCR products, Niles Pierce et al. developed digital HCR (dHCR), which limits product length to 200–300 nm through thermodynamic fine-tuning, enabling more precise single-molecule counting (Choi et al., 2018). Concurrently, by optimizing the reaction system and the concentration of hairpin-structured nucleic acids, as well as introducing modifications to the hairpin probes, researchers have controlled the length and homogeneity of the HCR, thereby preventing signal migration or fusion (Zheng et al., 2024). To address the mechanical instability of RCA products, George Church et al. significantly reduced signal drift by introducing modified bases (such as amino-allyl dUTP) during amplification and chemically cross-linking (e.g., BS(PEG)9) to anchor nanospheres *in situ* within a protein/nucleic acid matrix (Lee et al., 2014; Lee et al., 2015). Optical crowding is currently regulated primarily through two methods: physical expansion and biochemical regulation. STARmap, developed by Wang Xiao et al., and ExM, developed by Edward Boyden et al., utilize hydrogel embedding and physical expansion techniques, respectively, to increase the distance between molecules in three-dimensional space (Lee et al., 2014). Mats Nilsson et al. introduced cross-hybridized DNA oligonucleotides to induce tight folding, thereby reducing the physical scale (Clausson et al., 2015). Huang Yanyi et al. proposed a molecular dilution method in which nonprimer probes competitively occupy binding sites to reduce local fluorescence loading (Chang et al., 2023) (Table 3).

**TABLE 3:** Comparison of MERFISH, STARmap and HybISS.

Characteristics	MERFISH	STARmap	HybISS
Technical Principles	Image-based *in situ* hybridization	*In situ* sequencing, ISS	Sequence-by-hybridization ISS
Target Throughput	Full transcriptome scale (∼10,000 genes)	161 to 1,024	Detect tens to hundreds of target genes
Spatial Resolution	Subcellular/single-molecule resolution	Single-cell/Subcellular	Single-cell/Subcellular
Advantages	Extremely high detection efficiency and throughput; error-correction algorithms reduce the false positive rate; no enzymatic amplification required, with excellent penetration	Avoids the inefficient reverse transcription (RT) step in traditional *in situ* sequencing (*via* direct *in situ* ligation with SNAIL)	Using simple chemical hybridization (Seq-by-Hybridization) results in a higher signal-to-noise ratio and more stable readout

### 3.2 Specificity

In in situ nucleic acid visualization analysis, autofluorescence in the sample significantly affects the interpretation of the results. This is particularly true in formalin-fixed, paraffin-embedded (FFPE) tissue samples, where formaldehyde undergoes nucleophilic addition with lysine in tissue proteins to form a Schiff’s base. The broad emission spectrum produced under specific excitation light easily masks the true molecular signal (Zhang Z. et al., 2023; Baschong et al., 2001; Jia and Li, 2015; Ezeasor and Maina, 2025). Since FISH technology lacks a signal amplification mechanism, the target signal is often masked during the detection and analysis process. In addition, granular deposits of endogenous substances in the sample, such as lipofuscin, can easily cause confusion with the punctate signals generated by HCR and RCA (May-Zhang et al., 2022; Maynard et al., 2020). Furthermore, although the endogenous fluorescence of collagen fibers, elastic fibers, and hemoglobin in red blood cells results in distinct spatial morphological characteristics (such as distribution in the extracellular space), it still readily causes visual interference in the analysis of samples rich in connective tissue, such as the lungs and skin (e.g., ROS1 rearrangement detection) (Hsu et al., 2025; Pahlevaninezhad et al., 2014; Whittington and Wray, 2017; Conde et al., 2022). Currently, physical photobleaching or chemical pretreatment (such as Sudan Black B staining, sodium borohydride reduction, or hydrogen peroxide oxidation) are the mainstream methods for reducing such endogenous interference and improving detection sensitivity (Nolta et al., 2020; Schnell et al., 1999; Davis et al., 2014; Renier et al., 2014; Sapio et al., 2025).

The specificity of the technique itself fundamentally depends on the probe design and the rigor of the enzymatic reaction. FISH specificity is highly dependent on the sequence homology, CG content, and thermodynamic stability (Tm value) of the oligonucleotide probes (Beliveau et al., 2018; Hershberg et al., 2021). Among the software tools available for probe design, iFISH4U, ProbeDealer, and Tigerfish are particularly noteworthy. For routine probe design, which often requires highly specific single-copy probes, the iFISH4U platform employs K-mer segmentation, thermodynamic filtering, and alignment screening based on homology-weighted scoring to generate oligonucleotide probes with exceptional specificity (Gelali et al., 2019). ProbeDealer not only meets standard probe design needs but also supports the design of probes for highly multiplexed FISH. This platform allows for the flexible combination of probe backbone structures, such as the automatic and random addition of pre-designed barcode and primer sequences, which are accurately spliced to both ends of the target sequence. Additionally, the ProbeDealer platform simultaneously performs target sequence extraction, thermodynamic parameter filtering, secondary structure and hairpin structure prediction, as well as genome-wide BLAST off-target screening, significantly improving the efficiency of probe design (Hu et al., 2020). In studies involving the localization of repetitive sequences in the genome (such as centromeres, telomeres, and heterochromatin), traditional probe design algorithms often filter out these repetitive sequences, making probe design for these regions particularly challenging. Tigerfish is the first software specifically designed for designing Oligo-FISH probes targeting highly repetitive DNA regions; it can analyze combinations of region-specific short repetitive sequences. Using this software, the research team successfully designed probes targeting highly heterogeneous repeats on 24 different human chromosomes (Aguilar et al., 2024). The Tm value that is too low or too high can cause a shift in hybridization kinetics, leading to off-target or nonspecific binding of the probe (Ostromohov et al., 2018). During the probe design process, sliding-window approaches are typically combined with thermodynamic filtering. Additionally, the formation probabilities of secondary structures and hairpin structures are evaluated using appropriate tools, such as the online tools NUPACK and UNAfold (Zadeh et al., 2011; Zuker, 2003). Furthermore, the specificity of the probes is analyzed through comparison using online tools such as BLAST or Bowtie2 (Altschul et al., 1990; Langme et al., 2012) to minimize off-target binding.

In contrast, HCR and RCA introduce more complex logic gates at the technical level to reduce background noise. In the HCR v3.0 system, a unique reaction initiation scheme was established using a split-probe design, resulting in a 10- to 100-fold increase in specificity compared with that of HCR v2.0 (Choi et al., 2018; Schwarzkopf et al., 2021). Additionally, the Gibbs free energy of the hairpin-structured nucleic acid must be maintained at a low level at the reaction temperature (ΔG < −16 kcal/mol) to prevent spontaneous unwinding in the absence of an initiator strand (Li et al., 2020). Compared with the first two methods, RCA has the highest single-base specificity owing to the extremely high fidelity of the ligase in repairing the gap at the end of the lock probe, making it a core tool for single nucleotide polymorphism (SNP) detection. In addition to sequence design, the reaction system plays a crucial role in hybridization stability, including the effects of formamide concentration, ionic strength, and system viscosity on enzymatic kinetics (Yilmaz et al., 2012; Ku et al., 2004; Luby-Phelps, 2000).

### 3.3 Image processing

In the computational analysis of spatial transcriptomics of *in situ* sequencing, the conversion from raw cyclic optical images to single-cell spatial expression matrices currently represents the primary technical bottleneck. During the image preprocessing stage, multiround cyclic imaging is often accompanied by deformation and mechanical drift caused by tissue processing, which requires algorithms to perform subpixel-level elastic registration (Alon et al., 2021; Moses and Pachter, 2022; Schapiro et al., 2022). Additionally, in the highly heterogeneous solid tumor microenvironment (specifically within the necrotic core and dense stroma of tumors), tissue autofluorescence often causes traditional morphology-based background subtraction methods (such as top-hat filtering) to remove low-abundance signals, resulting in signal loss (Bahry et al., 2022; Eichenberger et al., 2021). Compared with image registration, the accuracy of cell segmentation is equally critical for the analysis of results. In dense tissues, traditional watershed expansion of the pseudocytoplasm—which relies solely on DAPI nuclear staining—struggles to anchor irregular cell membrane boundaries, leading to transcript crosstalk between adjacent cells (Li et al., 2024; Littman et al., 2021). To overcome these physical and morphological limitations, recent computational frameworks have begun to move away from purely image-driven segmentation logic. For example, the Baysor algorithm developed by Petukhov et al. innovatively incorporates a Markov random field (MRF) model to perform “image-free” molecular-level clustering segmentation directly using the resolved spatial coordinates and expression features of transcripts (Petukhov et al., 2022). The Mesmer deep learning framework proposed by Greenwald et al. achieves precise prediction of whole-cell boundaries in complex microenvironments through pantissue, multichannel feature fusion (Greenwald et al., 2022).

## 4 Clinical applications and translational research of in situ nucleic acid visualization technologies

### 4.1 Application of in situ nucleic acid visualization technologies in clinical research

The progression and immune evasion of solid tumors are highly dependent on their complex tumor microenvironment (TIME). Bassiouni et al. utilized spatial transcriptomics to analyze a diverse TNBC cohort, revealing a conserved spatial architecture of the tumor and precisely delineating regions of immune exclusion and immune enrichment (Bassiouni et al., 2023). With respect to brain gliomas, spatial sequencing was used to compare the microenvironments of primary tumors and brain metastases, revealing a remodeling of the spatial distribution of immune cells during metastasis (Motevasseli et al., 2024). Furthermore, *in situ* nucleic acid visualization technology has made significant contributions to the identification of tertiary lymphoid structures (TLSs). TLSs, as an ectopic lymphatic network, serve as independent biomarkers for predicting response rates to immune checkpoint inhibitors (ICIs). The integration of pathology with spatial transcriptomics has revealed specific chemokine networks within TLSs in gastrointestinal tumors, revealing that in CRC, CD16^+^ macrophages primarily recruit effector CD8^+^ T cells spatially *via* the CXCL16–CXCR6 axis (Yu R. et al., 2025). Nucleic acid *in situ* visualization technology also performs well in the identification of microbial infections. For example, in the analysis of HPV infection, this technology can be used to determine whether the HPV is in a free or integrated state, thereby aiding in the formulation of subsequent treatment plans (Wright et al., 2020). In the diagnosis of single nucleotide polymorphism, RCA-based *in situ* nucleic acid visualization technology has demonstrated excellent performance. For example, in the detection of point mutations in key driver genes (such as KRAS or BRAF) in solid tumors such as colorectal cancer, RCA technology enables the *in situ* differentiation of single-nucleotide variants (SNVs) with extremely high specificity without disrupting the structure of the tumor microenvironment (Larsson et al., 2010).

### 4.2 Potential clinical applications of in situ nucleic acid visualization technology

In the development of adoptive cell therapies (such as CAR-T or TCR-T) and neoantigen-based tumor vaccines, tracking the clonal expansion of specific T cells within the dense tissue of solid tumors remains a major challenge. RCA-based *in situ* nucleic acid visualization technology, with its single-base-pair specificity for *in situ* sequencing, holds the key to solving this problem. Groundbreaking advancements in 2025 led to the development of targeted RCA-based *in situ* T-cell receptor sequencing technology (*in situ* TCR-seq). The research team not only precisely read the CDR3 region sequences of the TCR αβ chains in tumor-infiltrating lymphocytes (TILs) *in situ* but also spatially anchored these clonal types to the expression of specific neoantigen mRNAs in adjacent cancer cells. This marked a significant increase in in situ nucleic acid visualization technology, moving beyond mere “phenotypic observation” to formally enter the realm of “genotype‒phenotype spatial correlation,” providing a reference for target validation in next-generation personalized immunotherapy (Yu H. et al., 2025).

At the level of algorithm and multimodal data integration, AI-driven analytical frameworks are transforming the logic of pathological diagnosis. By relying on multimodal AI, researchers have deeply integrated spatial transcriptomics, spatial proteomics, and whole-slide digital pathology images (WSIs) to systematically analyze the spatial heterogeneity of the tumor immune microenvironment driven by ancestry-related factors in early-onset colorectal cancer (EOCRC) (Pizurica et al., 2024; Wu et al., 2023). Furthermore, the next-generation MERFISH 2.0 platform prospectively integrates a deep learning-based, prior-free algorithm for segmenting true cell membrane boundaries when high-throughput tissue microarrays (TMAs) for lung and breast cancer are being processed. This algorithm effectively reduces the spatial misalignment (cross-talk) of transcripts in dense tissues, accurately converting complex high-dimensional molecular matrices into morphological views that pathologists can intuitively interpret, thereby significantly improving clinical slide-reading efficiency and the reproducibility of spatial diagnoses (Wang H. et al., 2025; Jones et al., 2025).

### 4.3 Clinical application potential of HCR and RCA and potential challenges in translation

In multiplexed target detection, HCR demonstrates excellent compatibility. For example, HCR-based *in situ* imaging of protein‒protein complexes, as well as the development of an extended HCR (utilizing 13 hairpin structures), has made it possible to amplify multiple low-abundance nucleic acid and protein signals simultaneously on a single section (Wang Y. et al., 2024). Derivative RCA technologies, such as asmFISH, combine cleavable probe conjugation with RCA and have achieved efficient *in situ* genotyping of 82 genes in the kidneys of diabetic mice (Xia et al., 2026).

However, HCR and RCA have yet to be widely adopted in routine clinical pathology testing, primarily because of the following systemic obstacles. First, FFPE samples result in poor permeation of macromolecular enzymes. Clinical samples, particularly FFPE samples, inevitably contain many cross-linked proteins (Koji, 2025; Fortner and Bucur, 2024). RCA relies heavily on high-fidelity ligases (such as Phi29 or PBCV-1 DNA ligase) (Schneider and Meier, 2017). A 2023 study on RCA testing for HPV mRNA revealed that large-molecule enzymes penetrate highly cross-linked FFPE tissue extremely slowly, leading to significant fluctuations in target capture efficiency and a high risk of false negatives (Rao et al., 2023). Second, the primary issues are reagent costs and clinical turnaround time (TAT), as clinical pathology requires reports to be issued within 24–72 h. The experimental workflow for RCA/HCR-based *in situ* nucleic acid visualization techniques is more cumbersome than that of FISH is, increasing the time required for testing. Additionally, because RCA requires enzymatic reactions, its reagent costs are high, hindering its clinical adoption.

## 5 Conclusion and prospect

This review introduces *in situ* nucleic acid visualization techniques based on various strategies, including FISH, HCR, and RCA. In comparison, FISH offers a more streamlined reaction system than HCR- and RCA-based *in situ* nucleic acid visualization techniques do, requiring only three steps: tissue pretreatment, hybridization, and washing. It is currently the primary method for the clinical detection of large-fragment DNA abnormalities. However, since it lacks a signal amplification mechanism, it is susceptible to interference from spontaneous fluorescence in samples, making it extremely difficult to achieve single-base resolution detection (single nucleotide polymorphism detection). *In situ* sequencing technologies based on FISH (seqFISH plus/MERFISH) achieve a target nucleic acid capture efficiency of 70%–90%, which is significantly higher than the 20%–30% capture efficiency of RCA-based *in situ* sequencing platforms. Additionally, they avoid signal crowding caused by physical steric hindrance and do not require complex enzymatic reaction systems. Notably, owing to the absence of signal amplification, the resulting fluorescent signal is relatively weak (RCA can amplify signals by 1,000–5,000 times), necessitating a more precise signal detection system. Furthermore, FISH-based *in situ* sequencing platforms require the predesign of complex probe libraries for targeted detection, whereas RCA-based *in situ* sequencing technologies, such as FISSEQ, can utilize random hexamer primers for *in situ* reverse transcription and RCA to achieve true *de novo* whole-transcriptome sequencing on tissue sections without the need for predesigned gene-specific probes (Lee et al., 2014; Yan et al., 2022). The primary advantage of HCR-based *in situ* nucleic acid visualization technology lies in its self-assembling signal amplification strategy, which offers superior tissue penetration compared with that of RCA, which relies on enzymatic reactions. However, the absolute quantification capabilities of HCR and the risk of background leakage associated with hairpin probes remain concerns that warrant attention.

### 5.1 Multiomics codetection

In complex disease models, obtaining only spatial RNA information is no longer sufficient to comprehensively elucidate the true functional state of cells. The development of *in situ* multiomics codetection of nucleic acids and other biomolecules (such as proteins, epigenetic modifications, and metabolites) facilitates a deeper interpretation of biological questions. For example, in highly heterogeneous solid tumors such as triple-negative breast cancer (TNBC), a single-omics approach cannot accurately characterize the immune evasion network; instead, *in situ* colocalization analysis of oncogenic transcripts, surface immune checkpoint antigens, and chromatin accessibility is needed. Furthermore, when the microenvironment of colorectal cancer invasion is elucidated, the simultaneous detection of specific RNA transcripts and metabolic microenvironmental factors (such as hypoxia or lipid metabolites) is crucial for revealing metabolic‒epigenetic crosstalk. To this end, Takei et al. developed MINA technology, which pioneered *in situ* cosequencing of chromatin assembly and RNA within single cells (Takei et al., 2021), whereas Liu et al.’s DBiT-seq method successfully achieved spatial colocalization of the transcriptome with dozens of proteins (Liu et al., 2020). Subsequently, Zhang et al.’s spatial-CUT&Tag method combined with RNA sequencing completely broke down the spatial barriers between the epigenome and the transcriptome (Zhang D. et al., 2023). Recently, Sinem K. Saka et al. further optimized orthogonal chemistry and multiplex imaging to construct a highly multiplexed *in situ* protein‒RNA multimodal platform (Saka et al., 2019). Compared to traditional enzyme-mediated *in situ* amplification reactions (such as RCA), SABER (Signal Amplification By Exchange Reaction) ingeniously utilizes a primer exchange reaction. By utilizing chain-displacement polymerases, a long single-stranded DNA tander (secondary probe) containing numerous repetitive binding sites is synthesized. These synthesized DNA tanders are then introduced into cells to hybridize with the primary probe bound to the target nucleic acid. Finally, extremely short, fluorescently labeled imaging probes are added for signal labeling. This technique offers advantages such as enzyme-free *in situ* hybridization, high signal intensity, and programmability (Kishi et al., 2019). In addition, the pSABER technology reported by Brian J. Beliveau et al., in 2024 is also noteworthy. Compared with SABER, pSABER technology utilizes scaffolds to recruit enzymes, triggering secondary exponential enzymatic reactions, and the signal is increased more than fivefold compared to SABER (Attar et al., 2025).

However, the most prominent technical contradiction in multiomics colocalization analysis lies in the irreconcilable nature of sample pretreatment. For example, highly sensitive RNA detection based on HCR or RCA often relies on potent proteinase K digestion of tissue to expose nucleic acids, but this irreversibly destroys most of the antigenic epitopes required for protein detection; furthermore, the spatial physical crowding resulting from high-density, multitarget codetection, as well as the computational integration of multimodal, cross-scale data, remain core bottlenecks hindering this technology. Currently, the main strategies for co-localization of DNA, RNA, and proteins include the HCR Pro technology and the oligonucleotide-conjugated antibody approach. The HCR Pro technology optimizes the chemical buffer, eliminating the need for proteinase K digestion during sample processing and allowing the reaction to be completed at room temperature. It provides excellent protection for protein components in tissue samples, creating favorable conditions for subsequent protein-nucleic acid co-localization (Choi et al., 2018; Crossland et al., 2025). In addition to the HCR Pro technology, the oligonucleotide-conjugated antibody approach—which replaces the fluorescent group on the antibody with a DNA barcode—ensures that proteins can be labeled with probes for colocalization detection after nucleic acid hybridization is complete (He et al., 2022).

### 5.2 Three-dimensional whole-tissue imaging

Traditional 2D tissue sections essentially represent a dimensional reduction in three-dimensional biological organs, resulting in the loss of information regarding spatial interactions along the Z-axis. Therefore, the development of *in situ* transcriptomic sequencing for three-dimensional whole tissues or even entire organisms represents a major direction for the advancement of spatialomics. First, tracking long-range cellular communication networks—such as elucidating neuroimmune projection axes within intact brain tissue—requires a complete 3D topological structure. Second, 2D sections cannot provide the continuous spatiotemporal coordinates necessary for reconstructing the lineage-specific trajectories of complex organs during embryonic development. For deep-seated solid tumors such as colorectal cancer and their liver metastases, mapping the complete 3D angiogenic architecture and physical remodeling features of the microenvironment is crucial for a profound understanding of intratumoral hypoxia gradients and drug delivery barriers. Currently, 3D *in situ* technologies are experiencing rapid growth. Chen et al. achieved the first complete 3D transcriptomic panorama reconstruction of a mouse embryo using highly automated serial section Stereo-seq (Chen et al., 2022). Wang et al. combined their upgraded STARmap PLUS technology with tissue clearing techniques to achieve whole-transcriptome-scale, section-free 3D *in situ* sequencing in intact mouse brains (Shi et al., 2023) and Glaser et al. utilized a novel nondestructive light-sheet microscope to significantly increase the imaging throughput of 3D spatialomics (Glaser et al., 2022). Vickovic et al.’s three-dimensional *in situ* barcoding technology has further overcome the limitations of optical read depth within thick tissues (Pleška and Vickovic, 2025). Despite these promising prospects, the future development of 3D *in situ* sequencing still faces numerous challenges. First, the physical deformation (contraction or expansion) caused by the tissue clearing process can easily lead to distortion of the true physical distance coordinates between cells. Second, in dense 3D extracellular matrices, ensuring uniform penetration of sequencing probes and macromolecular amplifiers to sufficient depths is extremely difficult. Third, 3D whole-organ multimodal imaging generates massive amounts of raw optical data (ranging from terabytes to petabytes), and the associated data storage, noise reduction, and development of truly fully automated 3D cell boundary segmentation algorithms represent the most pressing computational challenges currently facing the field.
